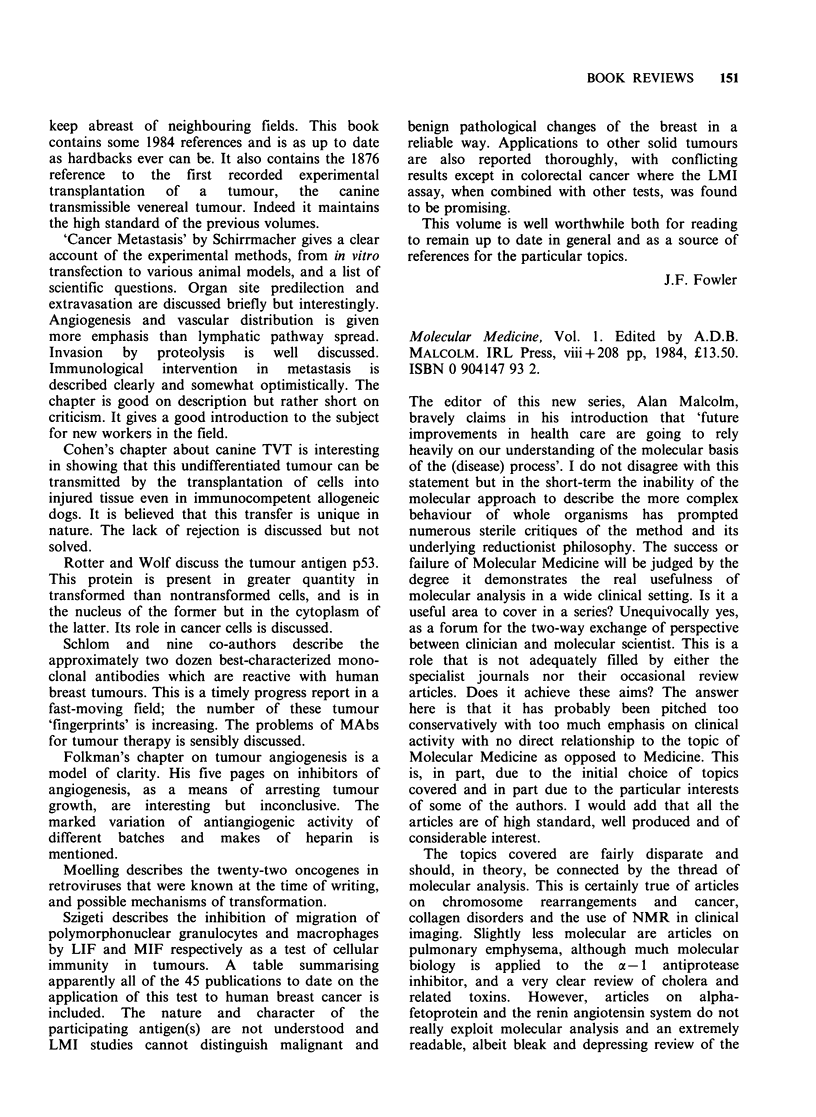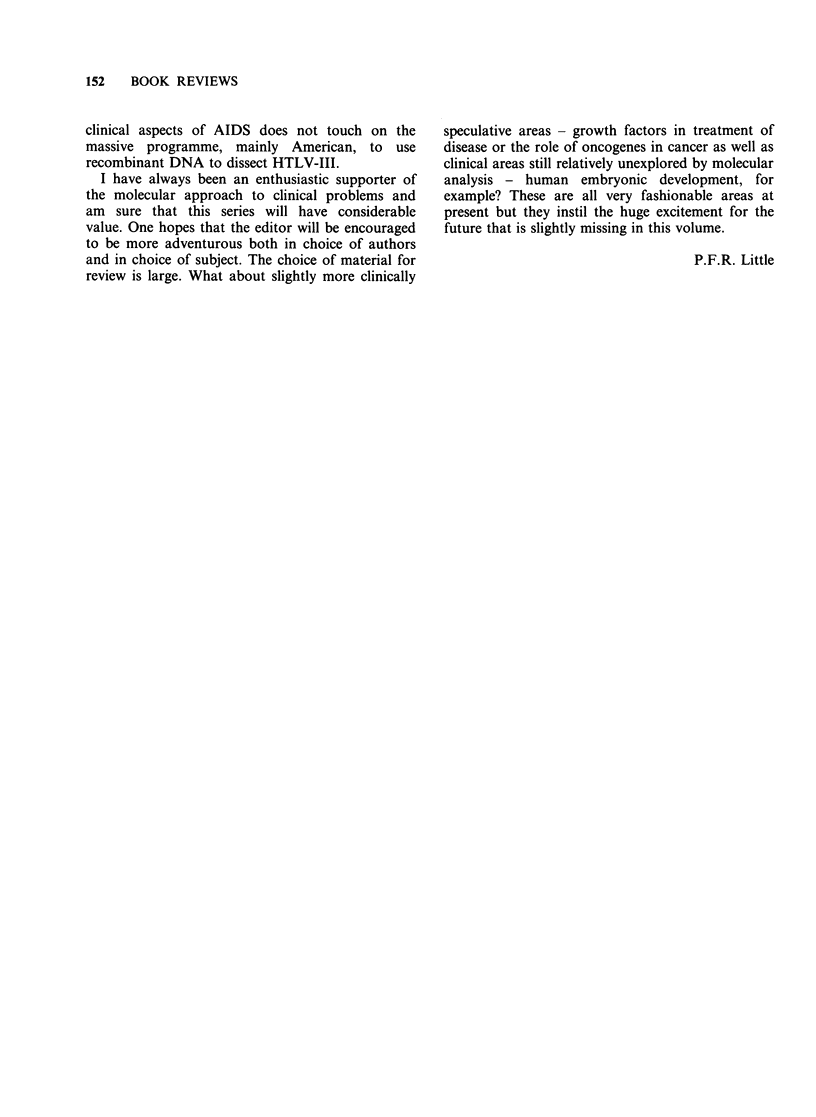# Molecular Medicine

**Published:** 1986-01

**Authors:** P.F.R. Little


*Molecular Medicine,* Vol. 1. Edited by A.D.B. MALCOLM. IRL Press, viii+208 pp, 1984, £13.50. ISBN 0 904147 93 2.

The editor of this new series, Alan Malcolm, bravely claims in his introduction that 'future improvements in health care are going to rely heavily on our understanding of the molecular basis of the (disease) process'. I do not disagree with this statement but in the short-term the inability of the molecular approach to describe the more complex behaviour of whole organisms has prompted numerous sterile critiques of the method and its underlying reductionist philosophy. The success or failure of Molecular Medicine will be judged by the degree it demonstrates the real usefulness of molecular analysis in a wide clinical setting. Is it a useful area to cover in a series? Unequivocally yes, as a forum for the two-way exchange of perspective between clinician and molecular scientist. This is a role that is not adequately filled by either the specialist journals nor their occasional review articles. Does it achieve these aims? The answer here is that it has probably been pitched too conservatively with too much emphasis on clinical activity with no direct relationship to the topic of Molecular Medicine as opposed to Medicine. This is, in part, due to the initial choice of topics covered and in part due to the particular interests of some of the authors. I would add that all the articles are of high standard, well produced and of considerable interest.

The topics covered are fairly disparate and should, in theory, be connected by the thread of molecular analysis. This is certainly true of articles on chromosome rearrangements and cancer, collagen disorders and the use of NMR in clinical imaging. Slightly less molecular are articles on pulmonary emphysema, although much molecular biology is applied to the $\alpha-1$ antiprotease inhibitor, and a very clear review of cholera and related toxins. However, articles on alpha-fetoprotein and the renin angiotensin system do not really exploit molecular analysis and an extremely readable, albeit bleak and depressing review of the clinical aspects of AIDS does not touch on the massive programme, mainly American, to use recombinant DNA to dissect HTLV-III.

I have always been an enthusiastic supporter of the molecular approach to clinical problems and am sure that this series will have considerable value. One hopes that the editor will be encouraged to be more adventurous both in choice of authors and in choice of subject. The choice of material for review is large. What about slightly more clinically speculative areas – growth factors in treatment of disease or the role of oncogenes in cancer as well as clinical areas still relatively unexplored by molecular analysis – human embryonic development, for example? These are all very fashionable areas at present but they instil the huge excitement for the future that is slightly missing in this volume.

P.F.R. Little